# Supplementary material for: Survey of Danish Head and Neck Cancer Patients’ Positions on Personalized Medicine, Gene Tests, and Personalized Follow-Up
Source: J Pers Med. 2024 Apr 11;14(4):404. doi: 10.3390/jpm14040404 (PMC11051027; doi:10.3390/jpm14040404)
Supplement: Supplementary file 1 [file jpm-14-00404-s001.zip › S2.pdf]

Supplementary material 2 (S2)

The full questionnaire data included a part of disease status (Part 0) completed by the examining physician.
The patients filled out the rest of the questionnaire. Hereof "Part 1" and "Part 2" represented the questions used from the survey by the Danish Ministry of Health and Danish Regions [5]. "Part 3" and "Part 4" were questions developed by the authors. The entire questionnaire was validated by semi-structured cognitive interviews.

Probe question:

Danish: "Efter at lave læst introduktion, vil du fortælle mig hvad du forstår ved "personlig medicin".
English: "After having read the introduction, tell me, with your own words your understanding of the concept "Personalized Medicine".

Full Questionnaire in Danish

Del 0: Udfyldes af lægen

Kræfttype

Mundhule ☐
Rhinopharynx ☐
Oropharynx ☐
Hypopharynx ☐
Larynx ☐
Spytkirtler ☐
Thyreoidea ☐
Sino-nasal ☐
Ukendt primær ☐

TNM

T: ______
N: ______
M:______

Kontroltidspunkt

2 mdr. ☐
3 mdr. ☐
6 mdr. ☐
12 mdr. ☐
18 mdr. ☐
24 mdr. ☐
36 mdr. ☐
48 mdr. ☐
60 mdr. ☐
Førtidskontrol ☐

Årsag for henvendelse:______________________________

Behandling

Kirurgi alene ☐
Kirurgi + Strålebehandling ☐
Kirurgi + Radioiod ☐
Strålebehandling alene ☐
Kemo-strålebehandling ☐
Kirurgi + Kemostrålebeh. ☐

Behandlingsstatus ______
(0: Ubehandlet tumor, 1: Aldrig tumorfri trods beh., 2: Intet recidiv efter prim. Beh., 3: Obs rediciv, 4: Ubehandlet recidiv, 5: Beh. for recidiv, aktiv sgd., 6: Beh. for recidiv, nu recidivfri)

Del 1 Demografiske data og uddannelse

1) Hvad er dit køn

   ☐ Mand
   ☐ Kvinde

2) Skriv venligst din alder: ___________

3) Hvilken kommune bor du i?

   ☐ Hjørring
   ☐ Brønderslev
   ☐ Frederikshavn
   ☐ Jammerbugt
   ☐ Læsø
   ☐ Mariagerfjord
   ☐ Morsø
   ☐ Rebild
   ☐ Thisted
   ☐ Vesthimmerland
   ☐ Aalborg
4) Hvad er din højest gennemførte uddannelse:

- ☐ Grund/folkeskolen (eller realeksamen)
- ☐ Gymnasial uddannelse (STX/HF/HH/HTX/HHX)
- ☐ Erhvervsfaglig uddannelse
- ☐ Kort videregående uddannelse under 3 år
- ☐ Mellemlang videregående uddannelse 3-4 år
- ☐ Lang videregående uddannelse 5 år eller mere
- ☐ Forskeruddannelse (f.eks. ph.d.)
- ☐ Andet: Angiv venligst:________________________

Del 2 Anvendelse af gen-test i sundhedsvæsenet

Derfor anvender man gen-test i sundhedsvæsenet.

Ved en gen-test ser man på menneskers gener og arvemasse, for eksempel DNA. Viden om gener og arvemasse kan bruges til at stille præcise diagnoser og til at udvikle mere effektiv behandling. Når gen-tests bruges til at skræddersy en behandling til en specifik patient, kaldes det ”personlig-medicin”.

Gen-test bruges allerede i dag på de danske hospitaler, for eksempel i kræftbehandlingen og til at rådgive om arvelige sygdomme. Der bliver hele tiden bedre muligheder for at forbedre behandling af sygdomme baseret på gen-test. En gen-test kan afsløre, at en patient har en øget risiko for at udvikle en bestemt sygdom. Den slags viden kan være nyttig. For eksempel hvis sygdommen kan forebygges effektivt. Men det er også en viden, som kan skabe bekymring. Uden gen-testen, ville patienten havde levet uden den bekymrende viden om øget risiko for sygdom. Det gælder særligt, når der er tale om en sygdom, som ikke kan forebygges.

1) Hvad tænker du umiddelbart om gen-test og personlig medicin efter, at du har læst introduktionen?

- ☐ 1 Det lyder spændende
- ☐ 2
- ☐ 3
- ☐ 4
- ☐ 5
- ☐ 6
- ☐ 7 Det lyder bekymrende

2) Hvor meget vurderer du, at du ved om gen-tests?

- ☐ 1 Ved ingenting om det
- ☐ 2
- ☐ 3
- ☐ 4
- ☐ 5
- ☐ 6
- ☐ 7 Ved rigtig meget om det

3) Har du nogensinde fået foretaget en gen-test (sæt eventuelt flere krydser)?

- ☐ Ja, jeg har selv opsøgt muligheden for at foretaget en gen-test
- ☐ Ja, jeg har fået foretaget en gen-test i forbindelse med behandling i det offentlige sundhedsvæsen
- ☐ Ja, jeg har fået foretaget en gen-test i forbindelse med et forskningsprojekt
- ☐ Nej, jeg har ikke fået en gen-test, men er det blevet tilbudt og sagde nej
- ☐ Nej, jeg har ikke fået foretaget en gen-test og er ikke blevet tilbudt det
- ☐ Ved ikke
- ☐ Ønsker ikke at svare

Gen-test bliver også brugt i forskningen, og her har man brug for information fra mange borgeres gen-test for at udvikle nye behandlingsformer. Det næste spørgsmål handler om, hvad du tænker om, at din gen-test anonymt kan bruges til forskning.

4) I hvor høj grad mener du, at det er vigtigt, at vi i Danmark forsker i gener, for at udvikle mere målrettet og personlig patientbehandling?

- ☐ 1 Slet ikke vigtigt
- ☐ 2
- ☐ 3
- ☐ 4
- ☐ 5
- ☐ 6
- ☐ 7 I meget høj grad vigtigt

5) Hvis din gen-test indgik i et forskningsprojekt og forskerne uventet opdagede, at du har en risiko for at udvikle en alvorlig sygdom, ville du så ønske at vide dette?

- ☐ Ja, lige meget hvad
- ☐ Ja, men kun hvis der findes en behandlings- eller forebyggelsesmulighed
- ☐ Nej
- ☐ Ved ikke

Del 3. Spørgsmål til personlig behandling, prognose og opfølgning efter kræftbehandling ud fra gen-tests. (Personlig Medicin i Kræftbehandling)

Nye detaljerede gen-tests kan muligvis finde spor af kræftcellers arvemateriale i enten blod- eller spytprøver samt detaljeret beskrive den enkelte kræftknudes opbygning på gen-niveau. Prøverne vil muligt også kunne bruges i et kontrolforløb til at opspore eventuelle tilbagefald tidligt.

1) Er du eller har du været tilfreds med det kontrolforløb, som er blevet planlagt til dig efter din kræftbehandling?

- ☐ Ja
- ☐ Nej
- ☐ Ved ikke
- ☐ Ønsker ikke at svare

2) Hvis du skulle lave om i dit kontrolprogram, hvad skulle der så være mulighed for? (Sæt eventuelt flere krydser)

- ☐ Færre planlagte kontroller på sygehuset
- ☐ Flere planlagte kontroller på sygehuset
- ☐ Færre planlagte scanninger
- ☐ Flere planlagte scanninger
- ☐ Færre eller ingen planlagte kontroller, men mulighed for kontakt til afdelingen og mulighed for at få en hurtig tid, hvis jeg oplever symptomer eller har bekymringer
- ☐ Jeg vil ikke lave noget om
- ☐ Jeg har ingen holdning hertil
- ☐ Andet, beskriv gerne____

The option (marked in red) "I would not change anything regarding my follow-up" in the abovementioned question was added following the cognitive interviews.

3) Hvis en blod- eller spytprøve med samme sikkerhed som en ambulant fremmødekontrol på sygehuset kunne fortælle, om der var tegn til tilbagefald, ville du så vælge blod- og spytprøver i stedet for de ambulante kontroller på sygehuset?

- ☐ Helt sikkert ikke
- ☐
- ☐
- ☐
- ☐
- ☐
- ☐ Helt sikkert

4) Hvis en blod- eller spytprøve taget hjemme eller hos egen læge havde en usikkerhed i forhold til at opdage et tilbagefald sammenlignet med en fremmødekontrol på sygehuset, hvor stor usikkerhed ville du så acceptere og stadig foretrække blod/spytprøven?

- ☐ Jeg ville aldrig vælge blod/spytprøven
- ☐ 1%
- ☐ 2%
- ☐ 5%
- ☐ 10%
- ☐ 20%
- ☐ Jeg vil ikke acceptere en usikkerhed (0%)

5) I dag har man kun få redskaber til at afgøre hvordan kræften vil reagere på en given behandling. Hvis man kunne udtale sig mere præcist om prognosen ved at undersøge kræftcellernes gener, ville du så ønske at få kendskab til denne prognose?

- ☐ Ja
- ☐ Nej
- ☐ Ved ikke

Del 4. Spørgsmål om svar på undersøgelser uden fysisk fremmøde

Denne del handler om de muligheder og udfordringer der kan være i forbindelse med personlig opfølgning via telefon eller per brev frem for fysisk fremmøde. Der vil med alle undersøgelser altid være en risiko for at man finder noget, man ikke forventede og dermed kan være nødt til at kontakte de involverede personer. Spørgsmålene handler primært om at få svar på undersøgelser, som man ved muligvis vil vise tegn på kræft, og de tanker der følger omkring at skulle modtage en sådan besked over telefon eller per brev.

1) Har du i forbindelse med udredningen, behandlingen eller opfølgningen af din kræftsygdom prøvet at få svar på undersøgelser uden at se lægen, dvs. over telefon eller per brev (sæt eventuelt flere krydser).

- ☐ Ja, planlagt svar på blodprøver
- ☐ Ja, planlagt svar på vævsprøver
- ☐ Ja, planlagt svar på scanninger
- ☐ Ja, ikke planlagt svar på undersøgelser på grund af ikke forventede fund
- ☐ Nej

2) Hvor stor en risiko for et dårligt svar vil du kunne acceptere hvis du med sygehuset skulle aftale at beskeden skulle gives uden fysisk fremmøde?

- ☐ Jeg ville aldrig vælge svar uden fysisk fremmøde, uanset risiko.

- ☐ 1%
- ☐ 2%
- ☐ 5%
- ☐ 10%
- ☐ 20%
- ☐ 50%

Full questionnaire in English

Part 0: Registered by the physician

Type of cancer

Oral cavity	☐
Rhinopharynx	☐
Oropharynx	☐
Hypopharynx	☐
Larynx	☐
Salivary gland	☐
Thyroid	☐
Sino-nasal	☐
Unknown primary	☐

TNM

T: ______

N: ______

M:______

Time from last treatment

2 months	☐	
3 months	☐	
6 months	☐	
12 months	☐	
18 months	☐	
24 months	☐	
36 months	☐	
48 months	☐	
60 months	☐	
Outside of planned schedule	☐	Reason:________________________________

Treatment

Surgery	☐
Surgery + Radiotherapy	☐
Surgery + Radioactive iodine	☐
Radiotherapy	☐
Chemoradiotherapy	☐
Surgery + Chemoradiotherapy	☐

Status (since last treatment) _____
(0: Untreated tumor, 1: Never free of tumor despite treatment, 2: tumor free after primary treatment, 3: Suspected recurrence, 4: Untreated recurrence, 5: Treated recurrence, active disease, 6: Treated recurrence, disease free)

Part 1 Demographic data and education

1) State your sex

- ☐ Male
- ☐ Female

2) Please state your age: __________

3) State your municipality?

- ☐ Hjørring
- ☐ Brønderslev
- ☐ Frederikshavn
- ☐ Jammerbugt
- ☐ Læsø
- ☐ Mariagerfjord
- ☐ Morsø
- ☐ Rebild
- ☐ Thisted
- ☐ Vesthimmerland
- ☐ Aalborg

4) What is your highest degree?

- ☐ Primary education
- ☐ General upper secondary education
- ☐ Vocational education
- ☐ Short-term higher education, below three years
- ☐ Medium-term higher education, three to four years
- ☐ Long-term higher education, five years or more
- ☐ Research education (Ph.D.)
- ☐ Other: Please elaborate: ______________________

Part 2 Use of gene test in healthcare

Why gene test is used in healthcare.
With a gene test you get information regarding people's genes and hereditary material (i.e., DNA). Knowledge about genes and hereditary material can be used to determine a precise diagnosis and

develop more efficient treatments. When gene tests are used to tailor a treatment for et specific patient, this is named "Personalized Medicine".

Gene tests are already used today at the Danish hospitals. Examples can be cancer treatment and to advise regarding genetic disorders. The possibility to improve treatment based on gene tests is continuously improving. A gene test can reveal if a patient has increased risk of developing specific diseases. This type of knowledge can be useful if the disease can be prevented effectively. But this type of knowledge can also be worrying. Without the gene test, a patient may live without having to worry about an increased risk of disease. This is especially important when it comes to the types of diseases that cannot be prevented.

1) Having read the introduction. What are your immediate thoughts about gene test and Personalized Medicine?

   - ☐ 1 Find It interesting
   - ☐ 2
   - ☐ 3
   - ☐ 4
   - ☐ 5
   - ☐ 6
   - ☐ 7 Find it worrying

2) How much do you know about gene tests?

   - ☐ 1 Know nothing
   - ☐ 2
   - ☐ 3
   - ☐ 4
   - ☐ 5
   - ☐ 6
   - ☐ 7 Know a lot

3) Have you ever had a gene test performed? (You can check multiple boxes)

   - ☐ Yes, I have sought out the possibility of a gene test on my own account
   - ☐ Yes, I have had a gene test performed during treatment in public healthcare
   - ☐ Yes, I have had a gene test performed as a part of a research project
   - ☐ No, I have not had a gene test performed. I have been asked, but I declined
   - ☐ No, I have not had a gene test performed. And I have not been asked
   - ☐ Unsure
   - ☐ Do not wish to answer

Gene tests is also being used in research and information from many people is needed to develop new treatments. The next questions are about your position gene test being anonymously used in research.

4) Is it important that we in Denmark perform gene research to develop more targeted and personalized patient treatment?

- ☐ 1 Not important at all
- ☐ 2
- ☐ 3
- ☐ 4
- ☐ 5
- ☐ 6
- ☐ 7 Very important

5) If you had a gene test performed as a part of a research project and the researchers unexpected discovered that you had a risk of developing a serious disease, would you like to get that information?
- ☐ Yes, no matter what
- ☐ Yes, but only if preventive or treatment options exist
- ☐ No
- ☐ Unsure

Part 3. Questions about personalized treatment, prognosis and follow-up after cancer treatment based on gene tests (Personalized Medicine in cancer treatment)

New advanced gene tests may be able to find traces of DNA from a cancer cell in either blood- or saliva samples. The test may also be able to describe the cancer more detailed. These samples could possibly also be used in the follow-up care and may detect recurrence early.

6) Are you, or have you been satisfied with the follow-up schedule that have been planned for you after your cancer treatment?

- ☐ Yes
- ☐ No
- ☐ Unsure
- ☐ Do not wish to answer

7) If you were to change anything in your follow-up, what would it be? (You can check multiple boxes)

- ☐ Fewer planned visits at the outpatient clinic
- ☐ More planned visits at the outpatient clinic
- ☐ Fewer planned diagnostic images
- ☐ More planned diagnostic images
- ☐ Fewer or no planned visits at the outpatient clinic but the possibility to contact the department and get an appointment as soon as possible if I have new symptoms or concerns
- ☐ I would not change anything regarding my follow-up
- ☐ I have no opinion regarding this
- ☐ Other, please elaborate:

  ____

The option (marked in red) "I would not change anything regarding my follow-up" in the abovementioned question was added following the cognitive interviews.

8) If a blood- or saliva sample with same accuracy as a physical examination in the outpatient clinic could reveal signs of recurrence, would you then choose the blood- or saliva samples over the physical examination in the outpatient clinic?

- ☐ Definitely not
- ☐
- ☐
- ☐
- ☐
- ☐
- ☐ Definitely

9) If a blood- or saliva sample performed at home or at your general practitioner performed inferior compared to a physical examination in the outpatient clinic, how large a difference would you accept?
- ☐ I would never choose the blood- or saliva sample
- ☐ 1%
- ☐ 2%
- ☐ 5%
- ☐ 10%
- ☐ 20%
- ☐ I would not accept any inferiority (0%)

10) Today, only few tools are available to determine how a cancer will react to the planned treatment. If an examination of the cancer cells genes could elaborate on the prognosis, would you like to receive this information?

- ☐ Yes
- ☐ No
- ☐ Unsure

Part 4. Question regarding receiving test results without physical attendance

This part concerns the possibilities and challenges regarding follow-up without physical attendance but via telephone or secure digital communication. All tests include a risk of unexpected findings and the subsequent need to contact the implicated persons. The questions are about how to receive test results during a workup for potential cancer and the concerns that may follow if such results are given via telephone or a secure digital communication.

5) Have you experienced to receive test results without physical attendance, i.e. via telephone or secure digital communication during your cancer evaluation, treatment or follow-up? (You can check multiple boxes).

- ☐ Yes, planned blood tests
- ☐ Yes, planned biopsies
- ☐ Yes, planned diagnostic images
- ☐ Yes, not planned answers due to unexpected findings
- ☐ No

6) How high a risk of a malignant/bad answer to a test result would you accept if you should agree to receive the result without physical attendance?

- ☐ I would never choose to receive test results without physical attendance. No matter the risk
- ☐ 1%
- ☐ 2%
- ☐ 5%
- ☐ 10%
- ☐ 20%
- ☐ 50%

References

5. Sundheds- og Ældreministeriet og Danske Regioner. *Danskernes Tanker Og Holdning Til Personlig Medicin Og Gentest*.; 2016. https://www.regioner.dk/media/4401/rapport-om-danskernes-holdning-til-personlig-medicin-og-gentest.pdf
