# Supplementary material for: Survey of Danish Head and Neck Cancer Patients’ Positions on Personalized Medicine, Gene Tests, and Personalized Follow-Up
Source: J Pers Med. 2024 Apr 11;14(4):404. doi: 10.3390/jpm14040404 (PMC11051027; doi:10.3390/jpm14040404)

Supplementary material for the manuscript "Survey of Danish Head and Neck Cancer Patients' Positions on Personalized Medicine, Gene Tests, and Personalized Follow-Up"

Supplementary material 3 (S3)

Bar Charts Showing the Distribution of Answers from the 177 Patients with Complete Data.

1)

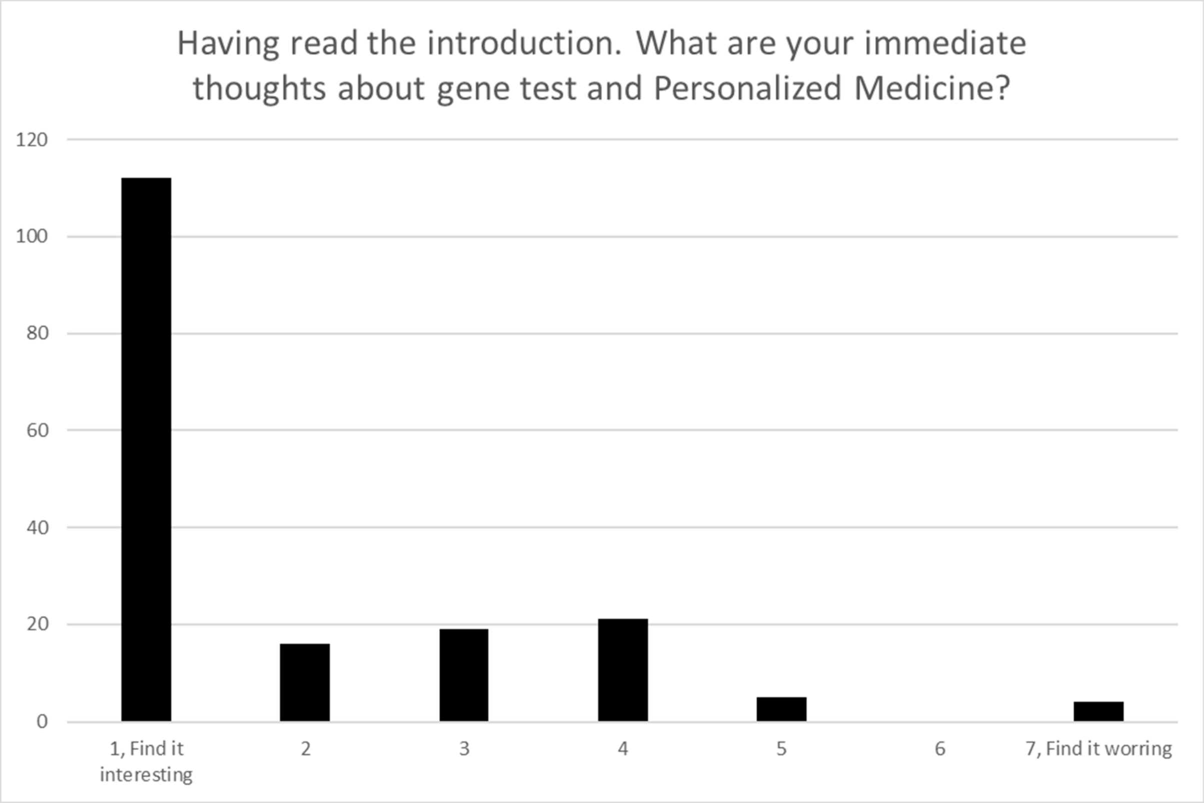

2)

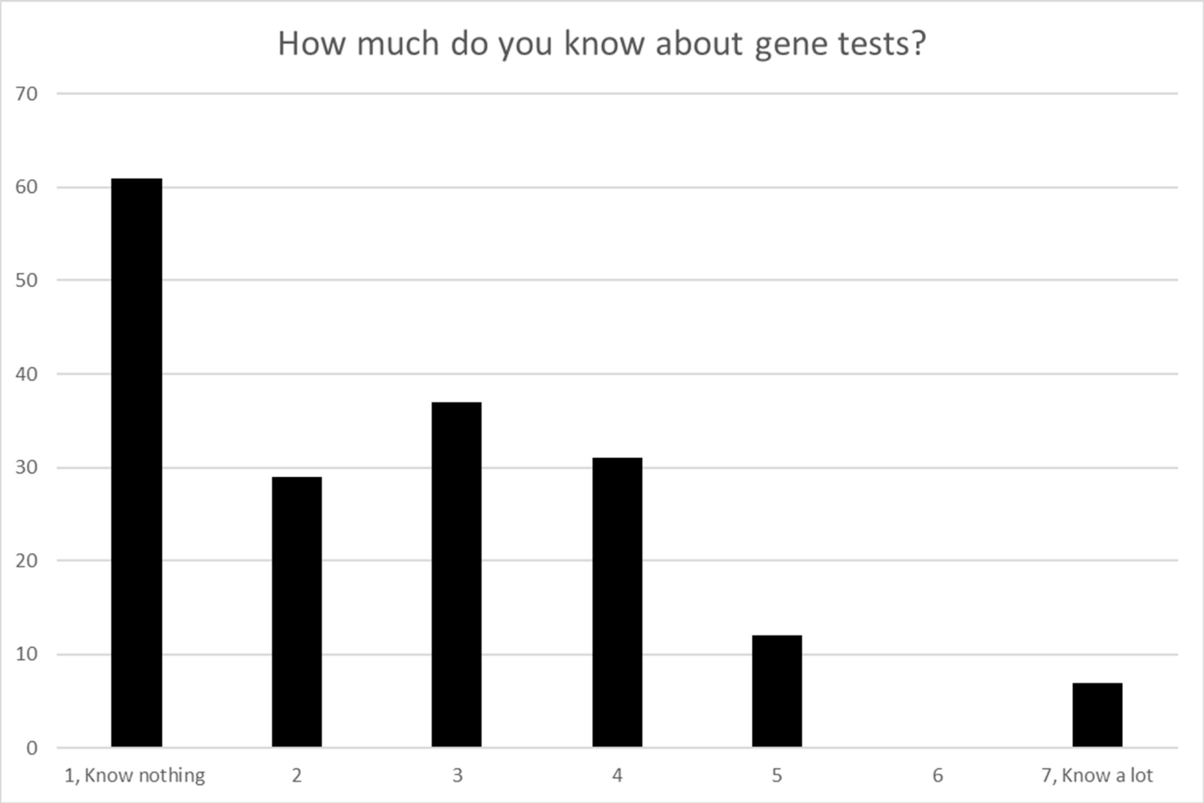

Supplementary material for the manuscript “Survey of Danish Head and Neck Cancer Patients’ Positions on Personalized Medicine, Gene Tests, and Personalized Follow-Up”

3)

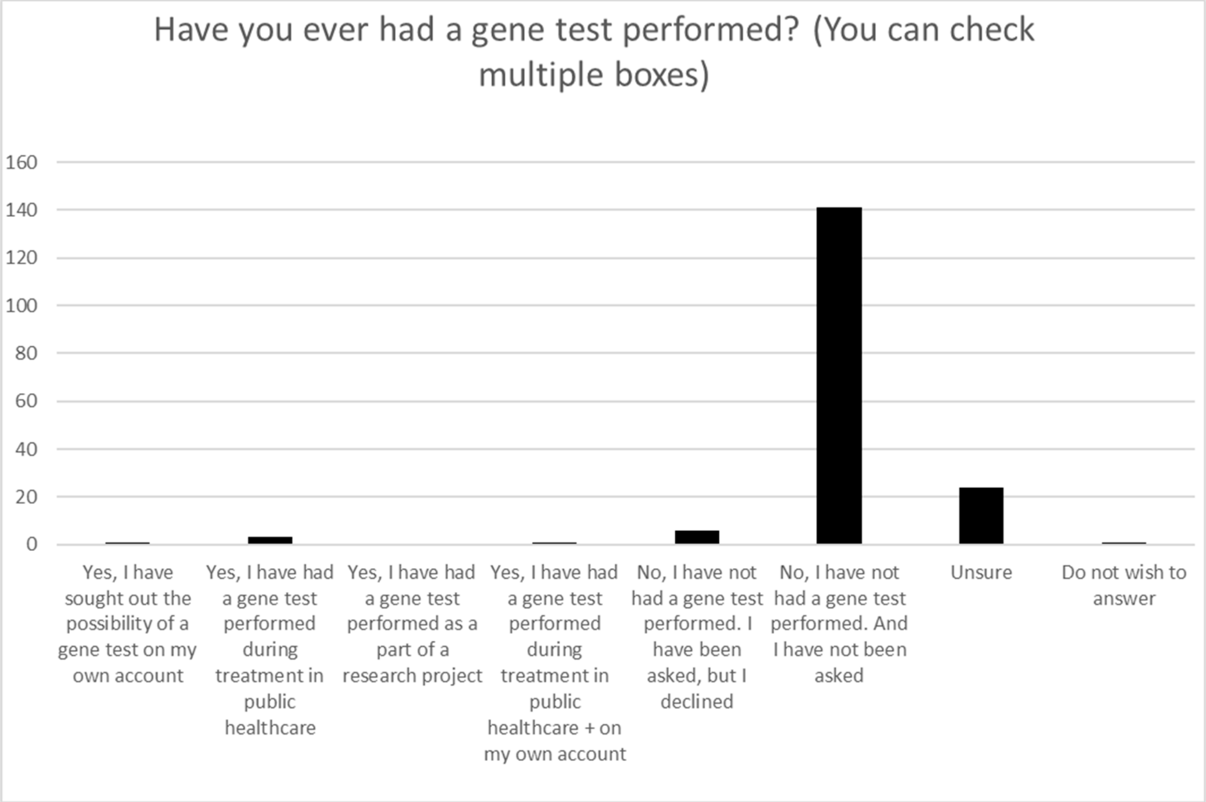

4)

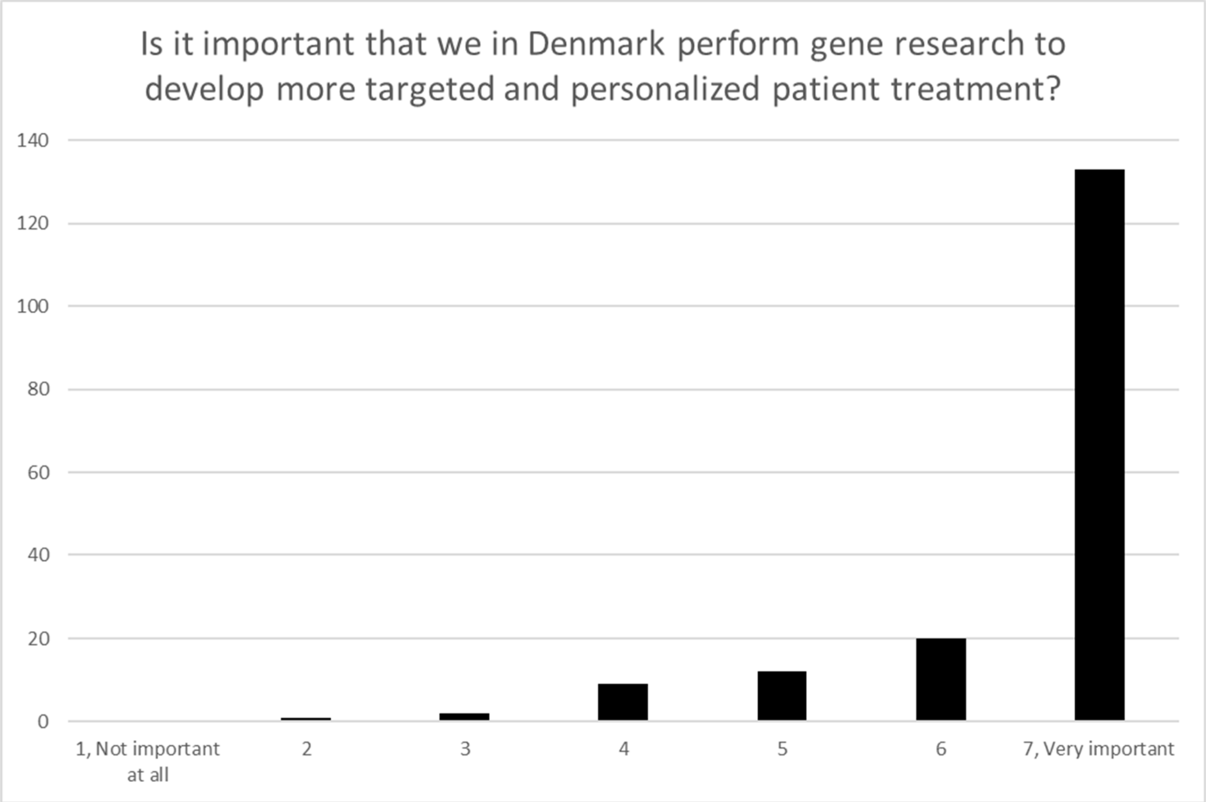

Supplementary material for the manuscript "Survey of Danish Head and Neck Cancer Patients' Positions on Personalized Medicine, Gene Tests, and Personalized Follow-Up"

5)

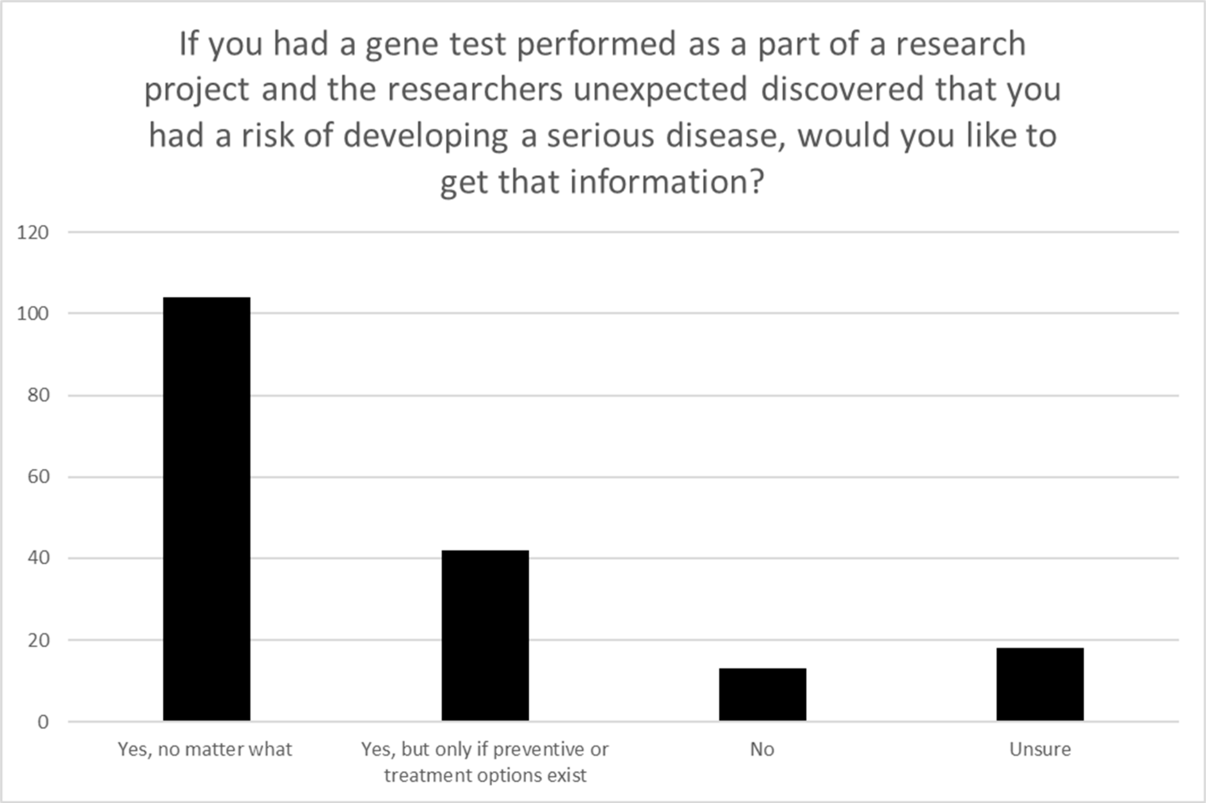

6)

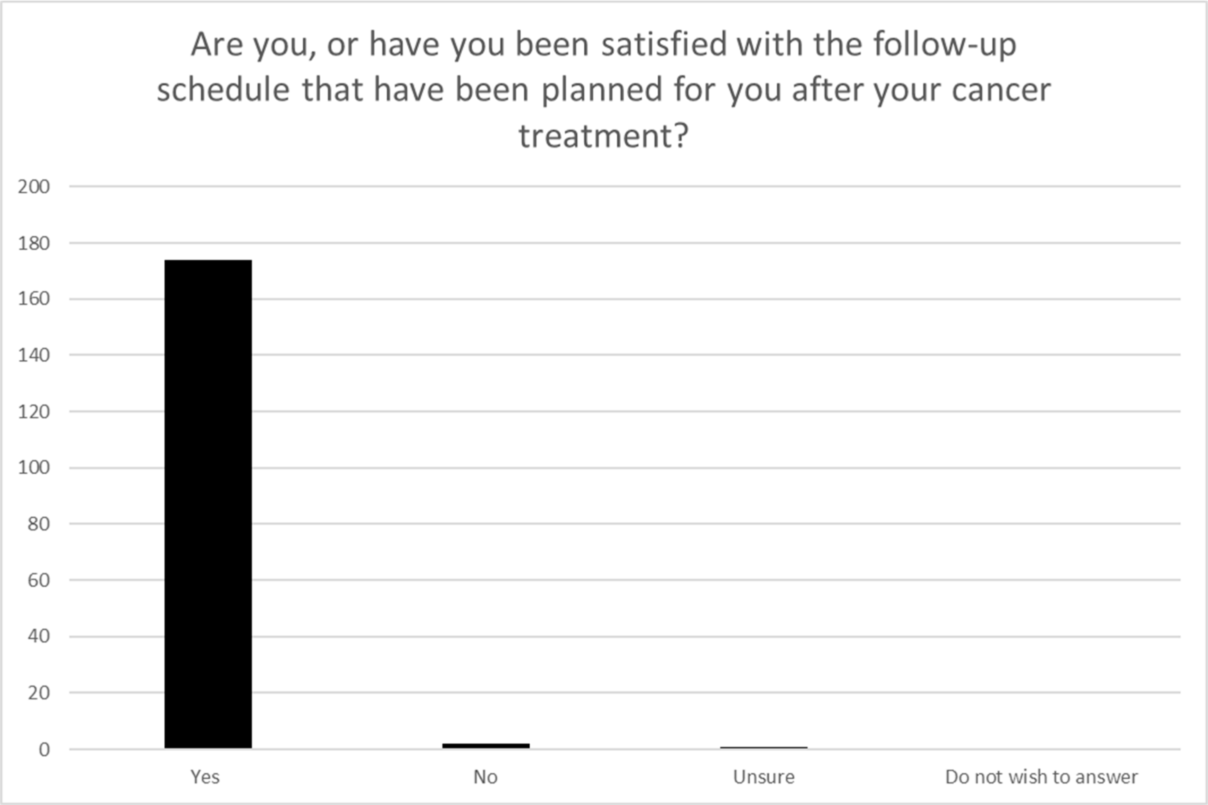

7)

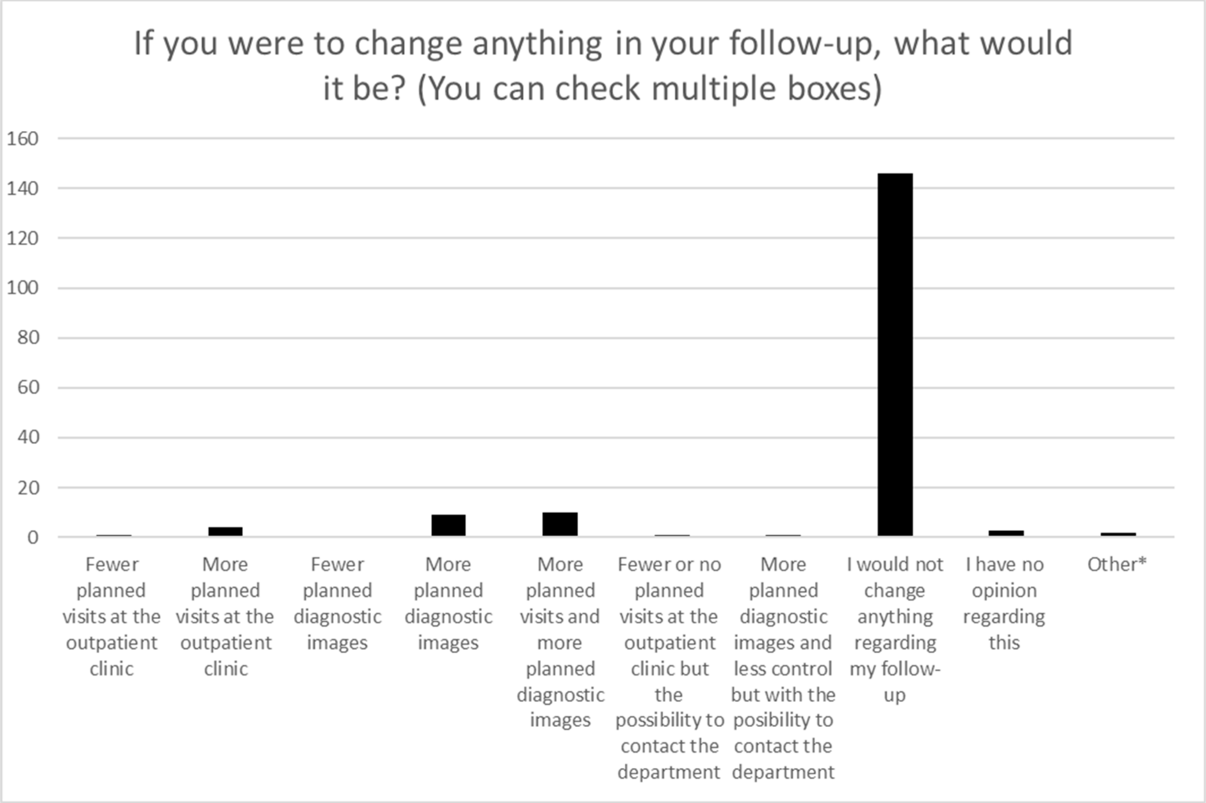

* One proposed longer follow-up period and one wanted better doctors.

8)

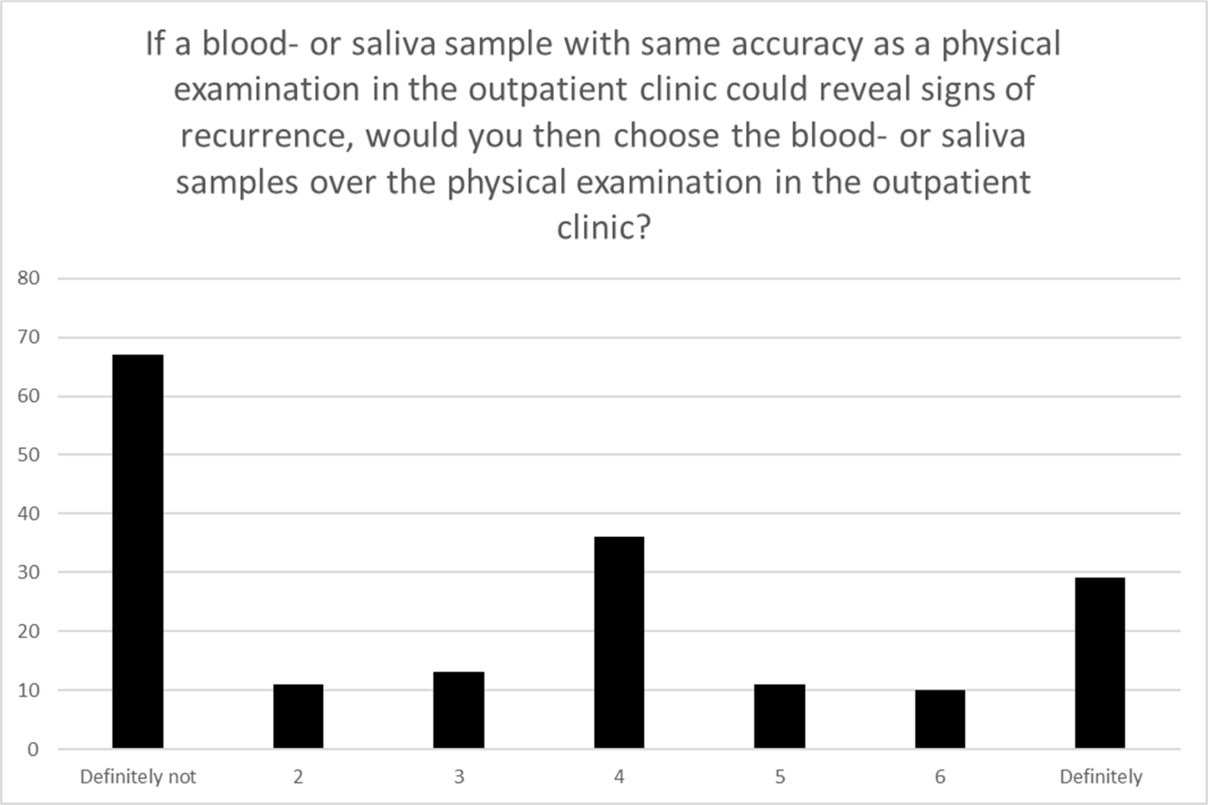

9)

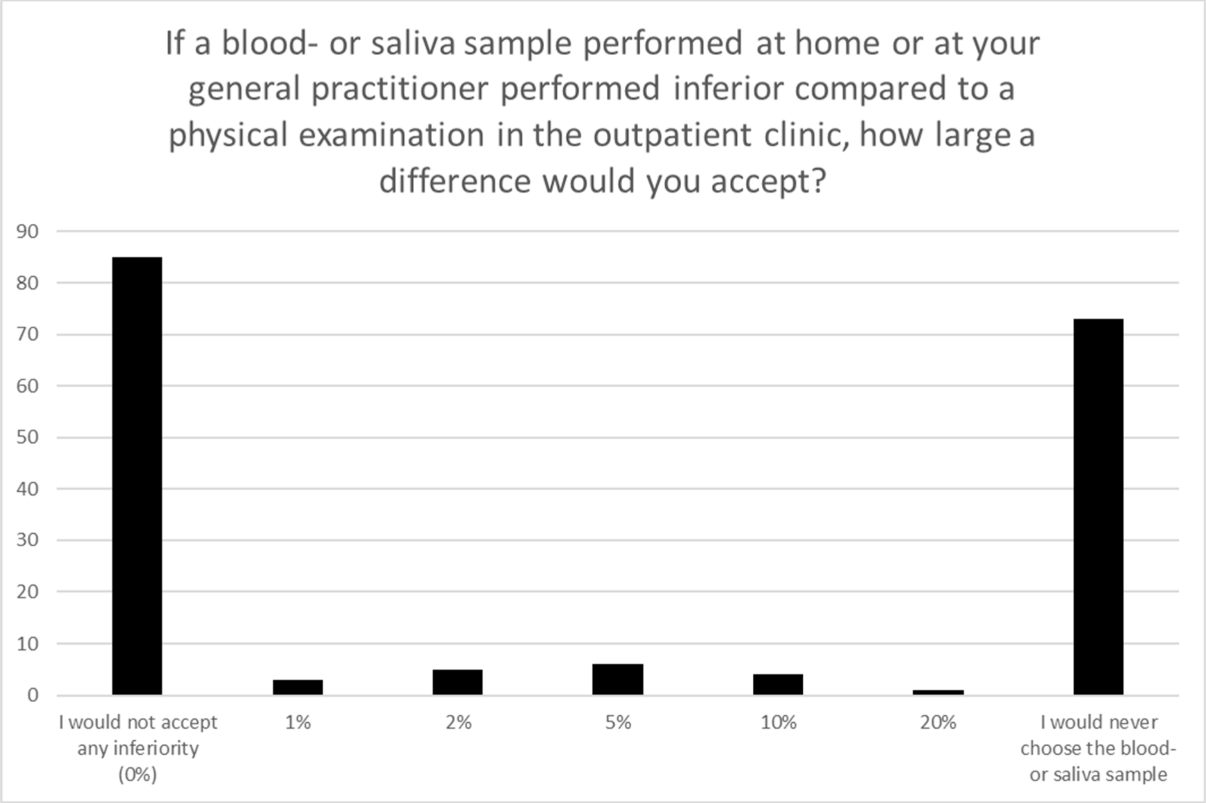
If a blood- or saliva sample performed at home or at your general practitioner performed inferior compared to a physical examination in the outpatient clinic, how large a difference would you accept?
90
80
70
60
50
40
30
20
10
0
I would not accept any inferiority (0%)
1%
2%
5%
10%
20%
I would never choose the blood- or saliva sample

10)

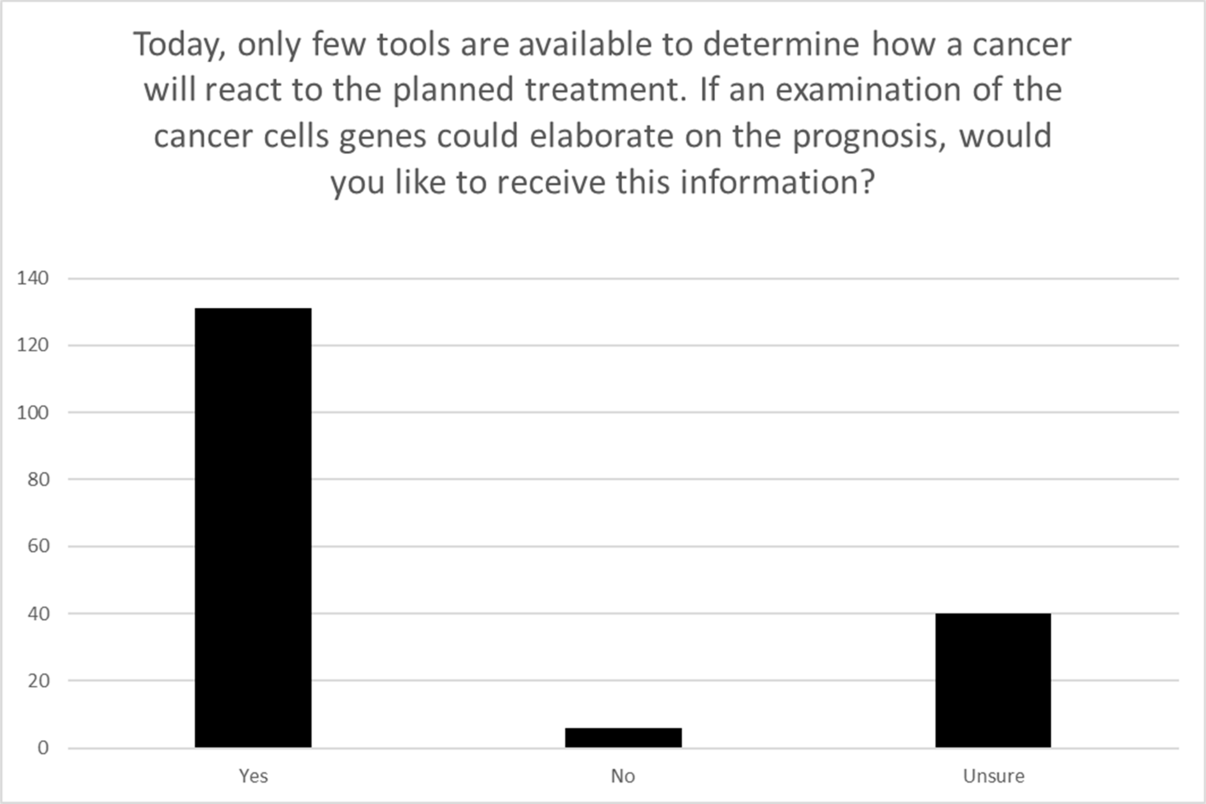
Today, only few tools are available to determine how a cancer will react to the planned treatment. If an examination of the cancer cells genes could elaborate on the prognosis, would you like to receive this information?
140
120
100
80
60
40
20
0
Yes
No
Unsure

Supplementary material for the manuscript "Survey of Danish Head and Neck Cancer Patients' Positions on Personalized Medicine, Gene Tests, and Personalized Follow-Up"

11)

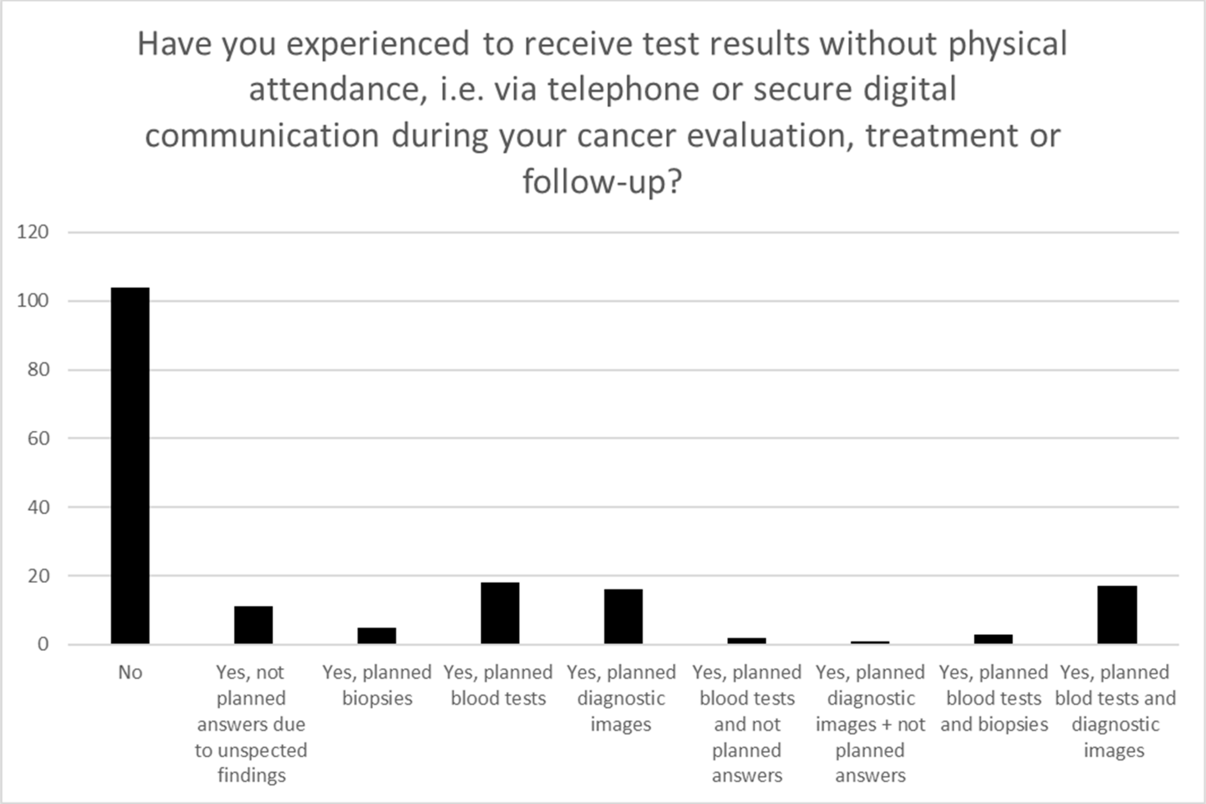

12)

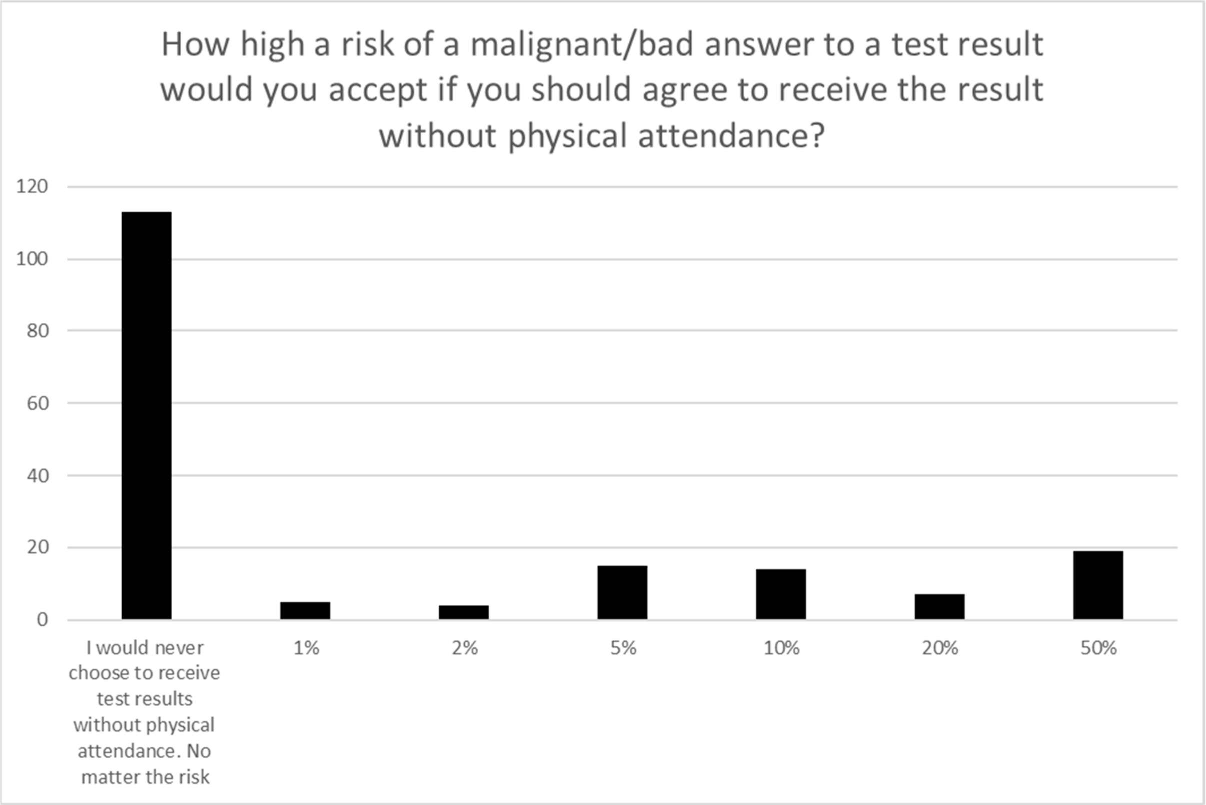

Supplement: Supplementary file 1 [file jpm-14-00404-s001.zip › S3.pdf]
